# 448. Real World Evaluation of Clinical Outcomes for Short-Duration Remdesivir Therapy in Hospitalized Patients with Mild-Moderate COVID-19

**DOI:** 10.1093/ofid/ofad500.518

**Published:** 2023-11-27

**Authors:** Kelsey N Williams, Timothy P Gauthier, Stacey W Baker, Jefferson Cua

**Affiliations:** Baptist Health South Florida, Fort Lauderdale, Florida; Baptist Health South Florida, Fort Lauderdale, Florida; Baptist Hospital of Miami, Miami, Florida; Baptist Health South Florida, Fort Lauderdale, Florida

## Abstract

**Background:**

The optimal duration of remdesivir for hospitalized patients with COVID-19 remains unclear. National Institute of Health (NIH) Guidelines recommend a standard duration of 5 days or until hospital discharge. This study aims to compare the outcomes of hospitalized patients who received ≤ 4 days of remdesivir compared to those who received ≥ 5 days.

**Methods:**

This retrospective study was conducted at a 900-bed community hospital from Jan 1 to Dec 31, 2022. Adult patients were included if ≥ 1 dose of remdesivir was received during hospitalization. Patients were excluded if hypoxic at baseline (oxygen saturation ≤ 94% or on supplemental oxygen). The primary outcome was COVID-19-related hospital readmission within 30 days. Secondary outcomes included hospital length of stay, 30-day mortality, and estimated healthcare cost avoidance (total drug cost and days of hospital admission avoided in the ≤ 4 day group).

**Results:**

1,253 unique patients were screened for inclusion. 772 were hypoxic at baseline, leaving 481 patients for analyses: 67 patients (14%) received ≥ 5 days and 406 (86%) received ≤ 4 days. Table 1 shows demographic data and baseline characteristics. COVID-19 related re-admissions within 30 days occurred in 2 (3%) patients in ≥ 5 day group and 6 (1%) in ≤ 4 day group (p=0.6). Median length of stay was 6 days in ≥ 5 day group and 4 days in ≤ 4 day group (p=< 0.0001). All-cause mortality within 30 days was 0 in the ≥ 5 day group and 5 (1%) in the ≤ 4 day group (p=0.61). No deaths were COVID-19 related. Hypoxia was observed during treatment in 25 (37%) in the ≥ 5 day group and 131 (32%) in the ≤ 4 day group (p=0.41); no difference in outcomes was observed between patients who did and did not develop hypoxia. Estimated cost savings in the ≤ 4 day group was $1,635,312.88 total ($426,312.88 in drug expenditure, $1,209,000.00 for 465 days of avoided admission), or $4,027.86 per patient. Outcomes data is available in Table 2.

**Figure:**
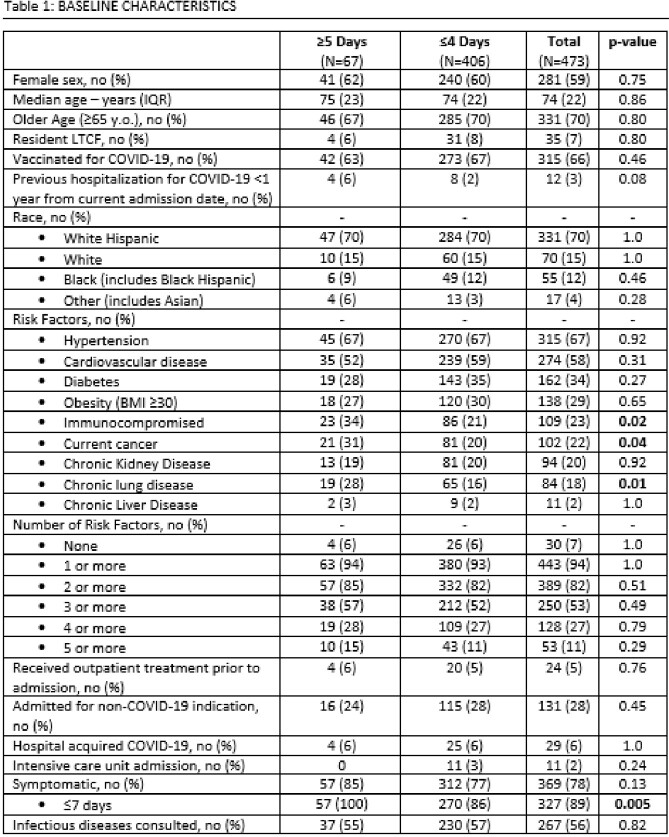

**Figure:**
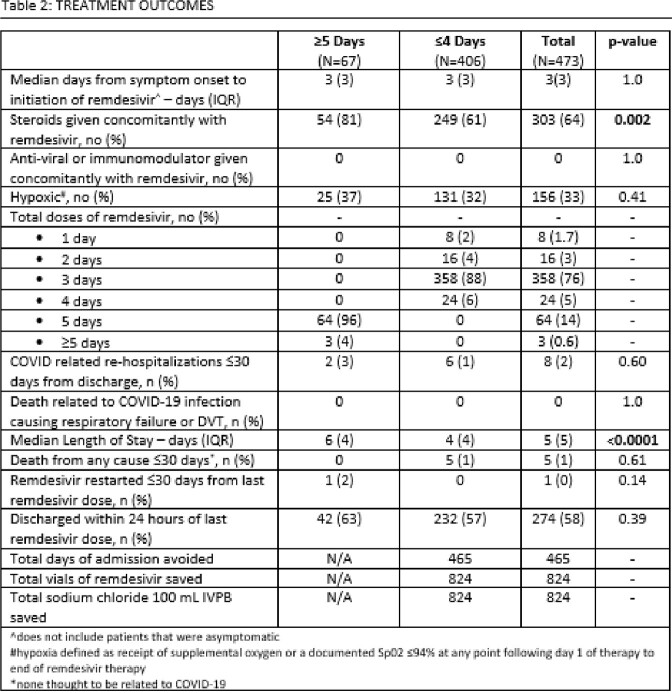

**Conclusion:**

Remdesivir for a full 5 days is not warranted in all at-risk hospitalized patients with mild-moderate COVID-19. Shorter remdesivir courses were not found to be associated with worse outcomes and had substantially reduced healthcare costs.

**Disclosures:**

**Timothy P. Gauthier, PharmD, BCPS, BCIDP**, Ferring: Advisor/Consultant|Firstline: Advisor/Consultant|Firstline: Writing|GSK: Advisor/Consultant|LearnAntibiotics.com & IDstewardship.com: Owner|Melinta: Advisor/Consultant|Pattern Biosciences: Advisor/Consultant|Pfizer: Advisor/Consultant

